# Optimal inference with suboptimal models: Addiction and active Bayesian inference

**DOI:** 10.1016/j.mehy.2014.12.007

**Published:** 2015-02

**Authors:** Philipp Schwartenbeck, Thomas H.B. FitzGerald, Christoph Mathys, Ray Dolan, Friedrich Wurst, Martin Kronbichler, Karl Friston

**Affiliations:** a The Wellcome Trust Centre for Neuroimaging, UCL, 12 Queen Square, London WC1N 3BG, UK; b Institute for Cognitive Neuroscience, University of Salzburg, Salzburg, Austria; c Neuroscience Institute, Christian-Doppler-Klinik, Paracelsus Medical University Salzburg, Salzburg, Austria; d Department of Psychiatry and Psychotherapy II, Christian-Doppler Hospital, Paracelsus Medical University, Salzburg, Austria

## Abstract

When casting behaviour as active (Bayesian) inference, optimal inference is defined with respect to an agent’s beliefs – based on its generative model of the world. This contrasts with normative accounts of choice behaviour, in which optimal actions are considered in relation to the true structure of the environment – as opposed to the agent’s beliefs about worldly states (or the task). This distinction shifts an understanding of suboptimal or pathological behaviour away from aberrant inference as such, to understanding the prior beliefs of a subject that cause them to behave less ‘optimally’ than *our* prior beliefs suggest they should behave. Put simply, suboptimal or pathological behaviour does not speak against understanding behaviour in terms of (Bayes optimal) inference, but rather calls for a more refined understanding of the subject’s generative model upon which their (optimal) Bayesian inference is based. Here, we discuss this fundamental distinction and its implications for understanding optimality, bounded rationality and pathological (choice) behaviour. We illustrate our argument using addictive choice behaviour in a recently described ‘limited offer’ task. Our simulations of pathological choices and addictive behaviour also generate some clear hypotheses, which we hope to pursue in ongoing empirical work.

## Introduction

### Optimal inference or optimal models?

Recent theoretical attempts to provide a unified understanding of behaviour and brain function in a dynamic and uncertain world cast cognition as probabilistic Bayesian inference [4,47,48,76], culminating in the Bayesian brain hypothesis ([27,36,37,54,65]. In this view, agents represent hidden states of the world as probability distributions and update their beliefs according to Bayes’ rule. Importantly, Bayesian inference is based on an agent’s generative model of the world, which is used to infer a probability distribution over causes (latent variables) generating sensory input. Formally, a generative model is a joint distribution *p*(*y*, *θ*) over sensory input (observations) *y* and subject-specific parameters *θ*, based on a likelihood function *p*(*y*|*θ*) and a prior distribution over parameters *p*(*θ*) [72]. Agents can then invert this model to find parameters that maximise the evidence of a given model in data, and thus infer the causal structure of their environment. Put simply, in order to make sense out the world, it is necessary to entertain a (generative) model of the causal structure that underlies noisy and constantly changing sensory impressions [23]. Therefore, an agent’s generative model crucially influences the result of an inference process.

Recent evidence, especially in the field of perception, suggests that the brain may indeed perform (close to) ‘optimal’ probabilistic inference [18,48,55,82,83]. In other instances, it has been shown that organisms substantially deviate from Bayes-optimality defined with respect to the true generative process [15,16,19,46,51], leading some to question whether humans truly perform Bayesian inference and suggesting that in many situations non-optimal models are employed; for example, simple heuristics when making decisions [17,43,52,77,78]. This question becomes particularly important when assessing individual differences in behaviour or characteristic aberrancies in psychopathology. For instance, how could a model based on ‘optimal’ Bayesian inference be used to account for characteristic suboptimal behaviour of a specific patient group?

Here, we propose two potential causes of suboptimal behaviour, which have radically different implications for understanding individual differences and psychiatric disease; namely, failures in inference and failures in the models upon which (optimal) inference is based (cf., [5]). We argue that patients showing pathological behaviour can still perform ‘optimal’ inference based on a particular generative model of the environment, which causes their behaviour to be less well adapted compared to control subjects. Importantly, the inference machinery itself is not broken, but instead a maladaptively constituted generative model causes pathological behaviour – based on (optimal) Bayesian inference. This implies a focus not on what patients perform in place of Bayesian inference but rather how their particular generative model – upon which inference is based – is constituted. Put simply, if the brain truly is a hierarchical Bayesian prediction machine, one can explain both normal and pathological behaviour. Identifying specific parameters or the specific constitution of the generative model in a patient group would have substantial impact on improving diagnosis, prediction of relapse or therapeutic success in clinical practise. Note that this marks a crucial advantage over approaches that focus on the deviation from optimality: investigating the subject-specific generative model of a task – as opposed to noting a subject’s deviation from ‘optimal’ behaviour – allows us (in principle) to identify the origins of aberrant inference in the cerebral hierarchy. In addition, this approach allows for a more nuanced perspective on the causes underlying observed behaviour. It is possible that some psychiatric conditions share characteristic aberrancies in expressed behaviour but that their particular causes are different. While noting the characteristic deviation from ‘optimality’ does not enable us to differentiate between different mechanisms that might cause this deviation, investigating the generative model underlying observed behaviour allows us to differentiate possible mechanisms that induce similar behaviour.

A prominent example, which we discuss in more detail below, is overly habitual and impoverished goal-directed behaviour both in addiction and obsessive compulsive disorders, which is likely to be induced by high impulsivity (low confidence) and high anxiety, respectively. It is important to note, however, that a computational approach to psychiatry rests on the assumption that some systematic aspects of the models used by a particular patient group can be identified, which may not always guaranteed in empirical work.

In summary, failing to account for the subject-specific generative models, means that there is only one way of acting optimally (e.g., by maximising the monetary gain in an economic decision-making task) and the only question one can pose is to what extent individuals differ from optimal behaviour as defined *a priori*. This is likely to create the impression that agents substantially deviate from Bayes-optimal inference and show significant inter-individual deviations. If one acknowledges individual variation in representing the environment, however, a second definition of optimality arises, which is relative to an agent’s generative model. In this framework, suboptimal or even pathologic behaviour is not understood as broken inference as such but as (optimal) Bayesian inference based on a suboptimal generative model of the world. Put differently, Bayesian inference based on individual models of the world will look suboptimal in a manner proportional to the differences between these models and our beliefs about the true structure of the world.

In the following, we discuss how inter-individual differences and psychiatric conditions can be understood in the context of Bayesian inference. To illustrate our argument, we consider a recently proposed Bayesian model of decision-making that casts choice behaviour as pure inference. We will briefly review the behavioural and physiological validity of this model and then discuss its application to translational work. Specifically, we will show how active inference can be used to characterise choice behaviour in patient cohorts (e.g., chronic alcohol abusers and pathological gamblers) using simulations.

### Normative or process models

Under the Bayesian brain hypothesis, a subject’s generative model characterises how a cognitive process can be performed by an embodied agent. In computational neuroscience one tries to understand how a certain problem could be solved in the brain to motivate a generative model of a task that might be implemented in the brain in a biologically plausible way.

Normative approaches try to understand cognition and behaviour with respect to optimising a particular quantity, such as maximising reward [74] or minimising surprise [38]. In such approaches, behaviour is governed by an overall principle that compels agents to optimise a specific quantity. Within such a general frameworks it is possible to formulate models with varying degrees of biological plausibility. For instance, it makes a substantial difference whether one simply describes an optimal solution to a task, such as a solution in a decision-making paradigm based on backwards induction and dynamic programming, or whether one takes into account the actual cognitive resources and neuronal message-passing necessary to perform a process given the computational resources of the brain [40]. In other words, normative approaches provide a state theory (about what the brain does), while biologically plausible implementations of the state theory furnish a process theory (about how it is done).

Characterising generative models allows one to formulate a process model of how cognitive processes are implemented in the brain, in contrast to models that rest on a correlational approach or are agnostic about the relationship between the mechanics of a model and brain functioning. The normative approach provides tools for assessing the extent of deviation from ‘optimal’ behaviour, given a model of the world that individuals or cohorts display. If normative theories are equipped with process models, they can provide direct insight into (maladaptive) computations that underlie pathology. Crucially, the question about the optimal solution to a task, given the world, becomes irrelevant because the real interest lies in the (optimal) solution, given an agent’s model of the world. ‘Bayes optimality’ can be conceived of as a principled way of performing optimally in a particular task but it can also be conceived of as optimal inference under an agent’s generative model of the world. When adopting the latter perspective, Bayesian inference can provide a process model of how agents actually behave – instead of a normative model of how they should behave.

We will see later that the most likely candidates for subject-specific differences in generative models are their prior beliefs. This is important because there are mathematical (complete class) theorems which suggest that any behaviour which can be described by a loss function can always be described as Bayes optimal under some prior beliefs. This means, that (at least mathematically) any ‘broken’ Bayesian inference, can always be characterised in terms of aberrant prior beliefs. This paper exploits this perspective to suggest that many neuropsychiatric conditions can be understood in terms of false inference under aberrant prior beliefs (including beliefs about beliefs, such as the confidence in – or precision of – one’s beliefs about the world).

### Individual differences and psychopathology in Bayesian inference

In cognitive neuroscience, we often try to understand inter-individual variability in cognitive processes and brain activity. Formally, this means that we (as observers) invert generative models of our subjects based on their observed responses (cf., [24]). This allows us to ask which parameters may have caused a distinct type of behaviour and enables us to investigate the relationship between specific parameters and personality traits or characteristics in behaviour, such as impulsivity or sensation seeking. This is the central procedure in the field of computational psychiatry [29,39,41,60,72].

There are several reasons why organisms might entertain generative models that ‘suboptimally’ approximate the true causal structure of the world. One reason is the accuracy-complexity trade-off inherent in Bayesian inference, as studied in machine learning [11,56]. To prevent overfitting of data, models have to optimise the trade-off between accurate predictions and generalizability, which means that good models have to restrict themselves from treating noise as meaningful information. Note that this is closely related to the concept of bounded rationality and satisficing as opposed to maximising [70,71]. Furthermore, specific circumstances may pose time pressure on behaviour, such as in classical fight or flight decisions. The use of parsimonious models may then become necessary because building a perfect model of the environment simply takes too long – or cannot accommodate slight changes in the environment. There could also be uncertainty about which model is best, inducing the need for Bayesian model averaging [35], or incompatible sensory data and priors; for example, in the case of perceptual illusions [42,53,79]. Also, non-ecological environments in experiments may lead to the adoption of inappropriate models since these may be the only models available to the agent based on her past experience. Most importantly, however, there may be phylogenetic constraints on the type of models that can be implemented biologically – as well as differences related to an individual’s ontogenetic development. The latter is of crucial importance for investigating pathologic behaviour – as it is possible that specific experiences in (biological) neurodevelopment may constrain subsequent models of the world, inducing, in extreme cases, a characteristic psychiatric condition. From a mathematical perspective, these considerations speak to approximate Bayesian inference in which the implementational problems of exact Bayesian inference are finessed using assumed forms for posterior beliefs. The particular choice of these forms speaks to the nature of the neuronal code and neuronal message passing within the brain. We will focus on a particular but ubiquitous form of approximate Bayesian inference later; namely, variational Bayesian updating through the minimisation of variational free energy – that provides a biologically plausible process theory.

In summary, characterising and individual’s generative model may be crucial when investigating group differences among different populations. Indeed, identifying particular characteristics of generative models associated with a particular psychiatric disorder marks a central aim of computational psychiatry [2,58,60,72]. An aim of computational psychiatry is to improve the diagnosis of psychiatric conditions and assess therapeutic progress. Assessing the characteristics of a generative model in a patient group allows one to investigate specific, trait-like characteristics of certain model parameters that may underlie pathological behaviour [72]. In doing so, researchers move from a purely descriptive account to understanding and explaining computational phenotypes that cause maladaptive conditions. Prominent examples for such approaches have been proposed in the field of autism [62], schizophrenia [1] or functional motor and sensory symptoms [31].

### Decision-making as active inference

As an illustration, we now present a model of choice behaviour in the framework of active Bayesian inference, and describe how abnormalities in this model might account for pathological decision-making in addiction. Crucially, to understand individual variability in choice, we estimated individual parameters of the generative model (of the task) based on observed behaviour, allowing us to identify specific parameters that may serve as biomarkers of behavioural and neuronal characteristics in substance-based and behavioural addiction, relative to a control group.

Recently, a generic model of decision processes has been proposed in which Markov Decision Problems (MDPs) are solved using (active) Bayesian inference based on variational (discrete-time) updates of prior beliefs about current states, action and control [40]. Crucially, agents try to minimise surprise about future states, where surprise is based on a belief-distribution about future states, in which desired states are more likely to be attained and undesired states the converse. Agents then infer an optimal policy by minimising the relative entropy or Kullback–Leibler-Divergence between their belief distribution and a distribution of likely outcomes. Intuitively, they increase the likelihood of visiting preferred states or goals by selecting policies that produce outcomes that are as close as possible to desired outcomes. Effectively, preferred outcomes are ensured by minimising surprise, where goals constitute the least surprising outcome. Crucially, not only beliefs about policies but also the precision (certainty) with which these beliefs are held have to be inferred. Precision can be understood as the confidence that a selected policy will be successful and thus controls the stochasticity or goal-directedness of an agent’s behaviour. Formally, precision is similar to the inverse temperature parameter in classical logistic or softmax choice rules [25,74]: however, there is a crucial difference – precision has a Bayes-optimal solution and has to be inferred and updated at each time-step. The resulting behaviour depends on a generative model of task contingencies and prior beliefs about precision – and therefore allows for an individual assessment of these model parameters and their relationship with behavioural tendencies, as illustrated in the following example.

**Fig. 1:**
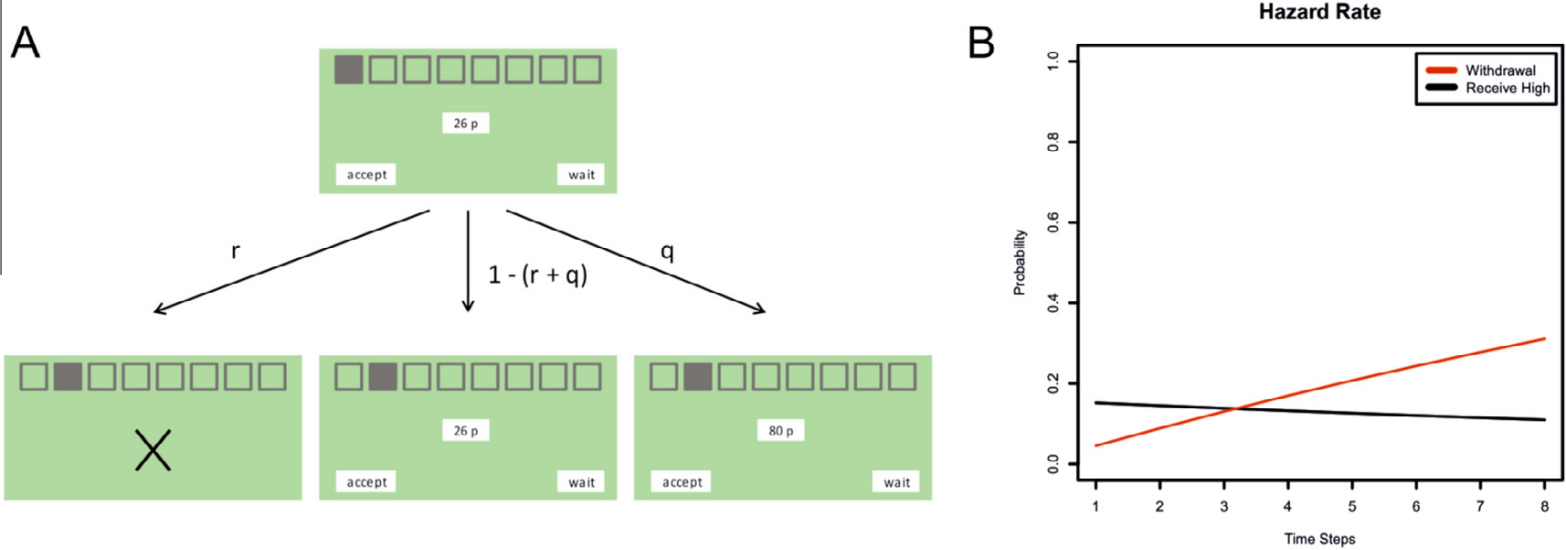
Task for model validation. (A) Each game comprised a specified number of trials (discrete time-steps). On each trial, subjects had to decide whether to accept a current offer or wait for the next trial. If subjects decided to wait, the low offer could be withdrawn with probability *r*, it could be replaced by a high offer with probability *q* or could be carried over to the next trial with probability 1 − (*r* + *q*). (B) The probabilities were defined by hazard rates, such that withdrawal probability increased, whereas a high offer became less likely the longer subjects waited. Adapted from [69].

We performed a behavioural and neuronal validation of this generic model in a functional magnetic resonance imaging (fMRI) experiment [69]. For this study, we developed a novel task in which participants had to infer the optimal time to accept an offer. In brief, subjects were presented with a small initial offer and had to decide how long to wait for a higher offer, with the risk of losing the initial offer and winning nothing (Fig. 1A). If they accepted too early, they precluded a higher offer at a later stage of the game, whereas if they accepted too late they risked losing the initial offer and winning nothing. To emphasise the context-sensitivity of this task, we defined the probabilities of receiving the high offer and losing the low offer as hazard rates, such that it became progressively less likely to receive the high offer and more likely to lose the initial offer (Fig. 1B). We compared long (8 trials) and short (4 trials) and varied the amount of the initial offer (between 9 and 35 pence, whereas the high offer was always 80 pence). In summary, subjects had to infer the optimal time to accept the initial offer taking the specific task contingencies into account, without waiting too long or not long enough.

**Fig. 2:**
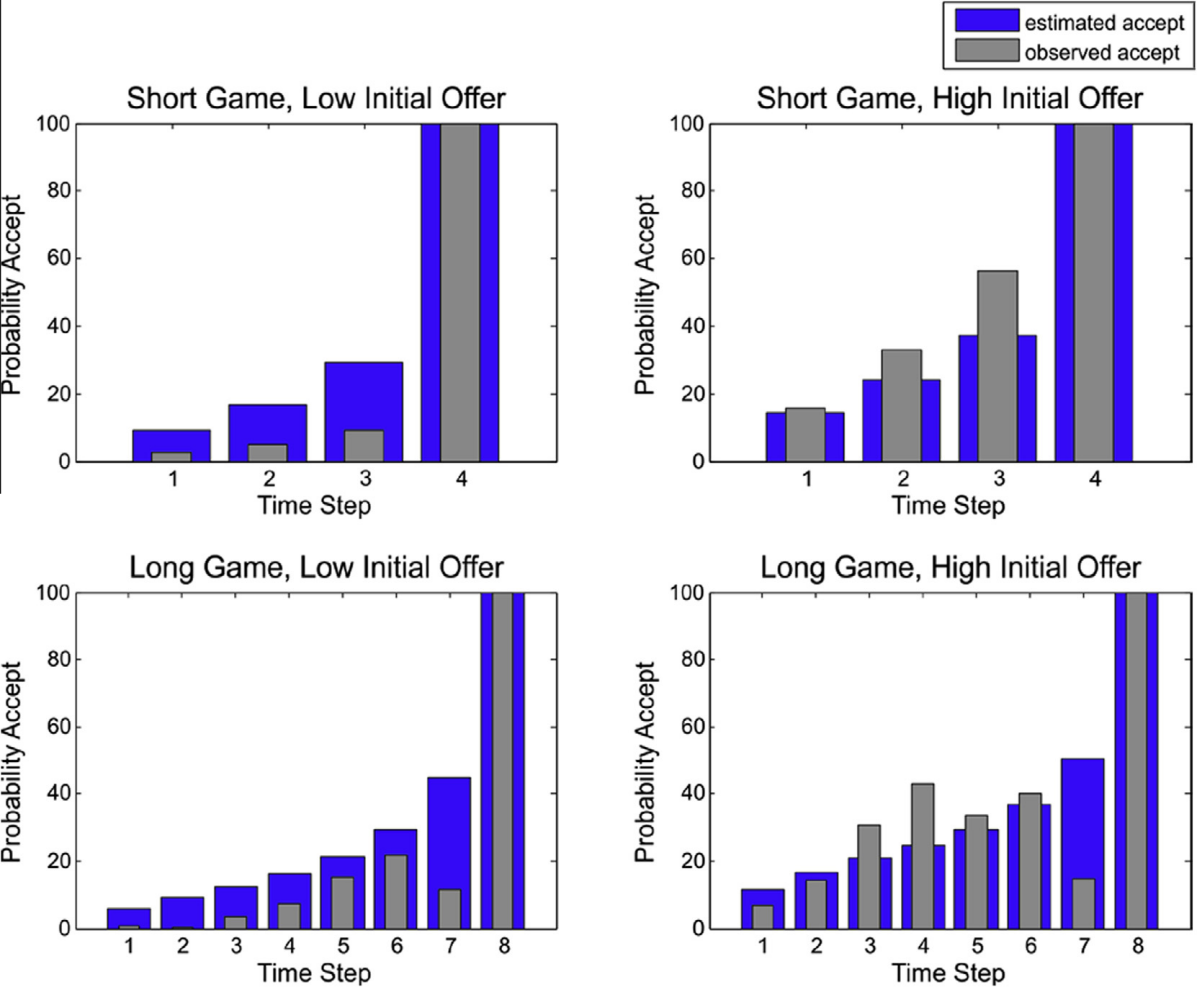
Results. Observed and estimated acceptance probabilities as predicted by our model (note that *P*_wait_ = 1 − *P*_accept_). These estimates are based upon maximum a posteriori values for prior precision, the hazard rate and sensitivity to differences in monetary cues.

We performed maximum a posteriori (MAP) modelling of task-relevant parameters (expected precision, hazard rate and sensitivity) by inverting the generative model of each subject, given observed behaviour. These parameters determined how a specific type of behaviour may have been caused. In brief, prior precision reflects the prior belief about the relative probability of competing policies or a “trait-confidence”. This means subjects with a higher prior precision are more confident about receiving the high offer if they deliberately wait. Estimates of expectations of hazard rates tell us how well subjects represented the task contingencies, whereas the sensitivity for the difference in utilities (log likelihood) of outcomes provides important information about how much subjects valued one outcome over others (see Fig. 2 for observed behaviour and model predictions).

Therefore, we were able to characterise the individual generative model (representation of the task) employed by each subject, upon which variational Bayesian updates were based. For instance, we found evidence that subjects who had higher prior precision accepted the initial offer less frequently and waited longer for the high offer. Furthermore, we were able to show that the updates of expected precision – based on the individual estimates of prior precision – were encoded by dopaminergic midbrain regions.

Having established the validity of this (active Bayesian inference) model of subject responses in behavioural and neuronal terms, we now ask whether it can be used to understand and classify (pathologic) choice behaviour in addiction. Specifically, we can now look in detail at how changes in a subject’s prior beliefs will produce characteristic patterns of behaviour by simulating choice behaviour in the limited offer game, under different prior expectations.

## Hypothesis and evaluation: addictive choice behaviour as optimal inference

In the following, we discuss characteristic abnormalities of behaviour in addiction and how these could be accounted for by an active inference model of choice behaviour. We will use simulations to illustrate maladaptive choice behaviour in our limited offer paradigm in both a qualitative and quantitative sense. These simulations generate some specific predictions, which we are currently testing empirically – using both behavioural and physiological responses.

Addiction is associated with characteristic patterns of maladaptive choice behaviour [49,61,63], and thus appears to be ideally suited for a computational approach to understand the basis of aberrant decision processes. Individuals with addiction have been found to be more impulsive [3,6,32], and neuronal activation associated with impulsivity or response inhibition can be used to predict future substance abuse [57]. Furthermore, addictive choice behaviour has been related to steeper delay discounting [59,64,66,73] – with the implication that the negative future consequences of addictive behaviour are strongly devalued – as well as to reduced risk sensitivity and decreased inhibitory control [21,20,50].

From a computational perspective, it has been argued that addictive behaviour may be caused by a shift from action-outcome to stimulus–response control [29], resulting in a predominance of less flexible, habitual modes of behaviour [32,45,67]. This has also been framed as the gradual transition from ‘impulsion to compulsion’, implying that addictive behaviour is marked initially by seeking hedonic pleasure from a certain substance intake or behaviour, whereas later this addictive behaviour becomes habitual and automatic [33]. Other perspectives invoke a shift from a predominance of liking to wanting in the context of incentive salience [8,9], implying that addicts show compulsive ‘wanting’ of drugs or drug-like behaviour without actually ‘liking’ it [68].

In addiction research there are several hypotheses that explain addictive behaviour. The impulsivity hypothesis states that individuals with addiction are more impulsive and worse at inhibiting a response than controls, which – combined with an increased sensitivity to reward – may induce addictive behaviour [3,10]. A reward-deficiency hypothesis, on the other hand, suggests that addicted individuals are less sensitive to nondrug rewards relative to drug-related rewards, which compels them to seek drugs [12]. Imaging studies provide evidence for both accounts [50]. Specifically, addictive behaviour has been associated with changes in reward processing [59,64] that is often associated with a dysfunction of the dopaminergic system [7,28,30,75] – for which there is also genetic evidence [13,14,22]. Most prominently, addiction is marked by a loss of phasic response of the dopaminergic system [80,81], which resonates with the reward-deficiency hypothesis – as this implies that only drug-related behaviour can elicit phasic dopaminergic responses and thus the feeling of reward.

**Fig. 3:**
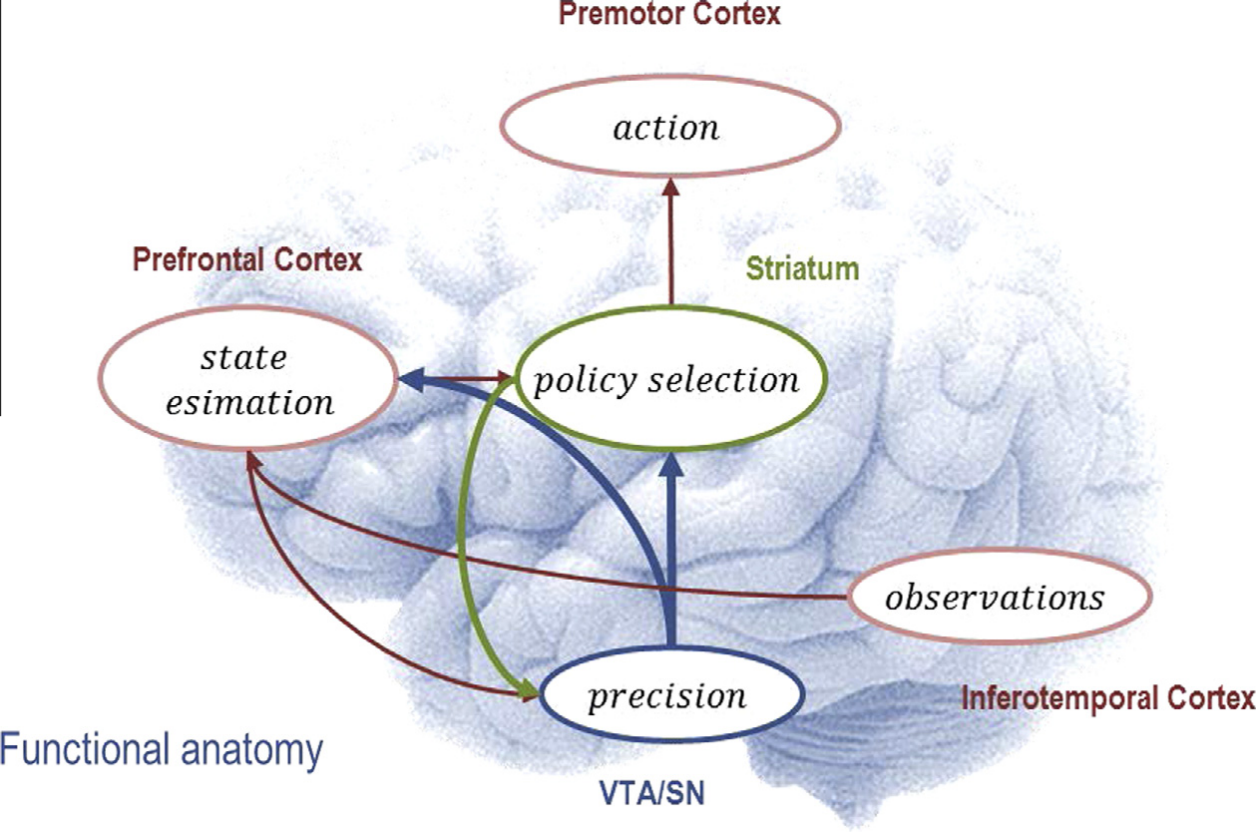
Proposed variational message passing scheme. This figure illustrates the proposed message passing scheme in active inference and its putative mapping to brain function. The scheme rests on variational updates, such that several distinct representations (expectations of states, polices and precision) are updated and inform each other – providing a nice metaphor for functional segregation and integration in the brain. Here, current observations inform inference about hidden states of the environment, which induce updates of precision. Precision influences policy selection which in turn specifies actions – that are sampled from the most likely policies. Note that the connections are recursive because variation updates require a reciprocal exchange of sufficient statistics. Recent empirical work has shown that expected precision is encoded in the dopaminergic midbrain [69].

In summary, addiction appears to be strongly related to impulsivity and a lack of inhibitory control, a preponderance of habitual as opposed to goal-directed or planned behaviour and a hypersensitivity to rewarding, drug-related stimuli. Given the large body of empirical evidence for these characteristics of addictive (choice) behaviour – what can we gain from an active inference perspective on addiction? As discussed earlier, the important question is whether specific parameters (in subject-specific generative models) can be identified that explains group differences in addictive compared to normal choice behaviour: in other words, what sorts of generative model (or model parameters) produce (Bayes optimal) addictive behaviour? Fig. 3 provides an intuitive depiction of the proposed variational message passing scheme in active inference upon which our considerations are based.

**Fig. 4:**
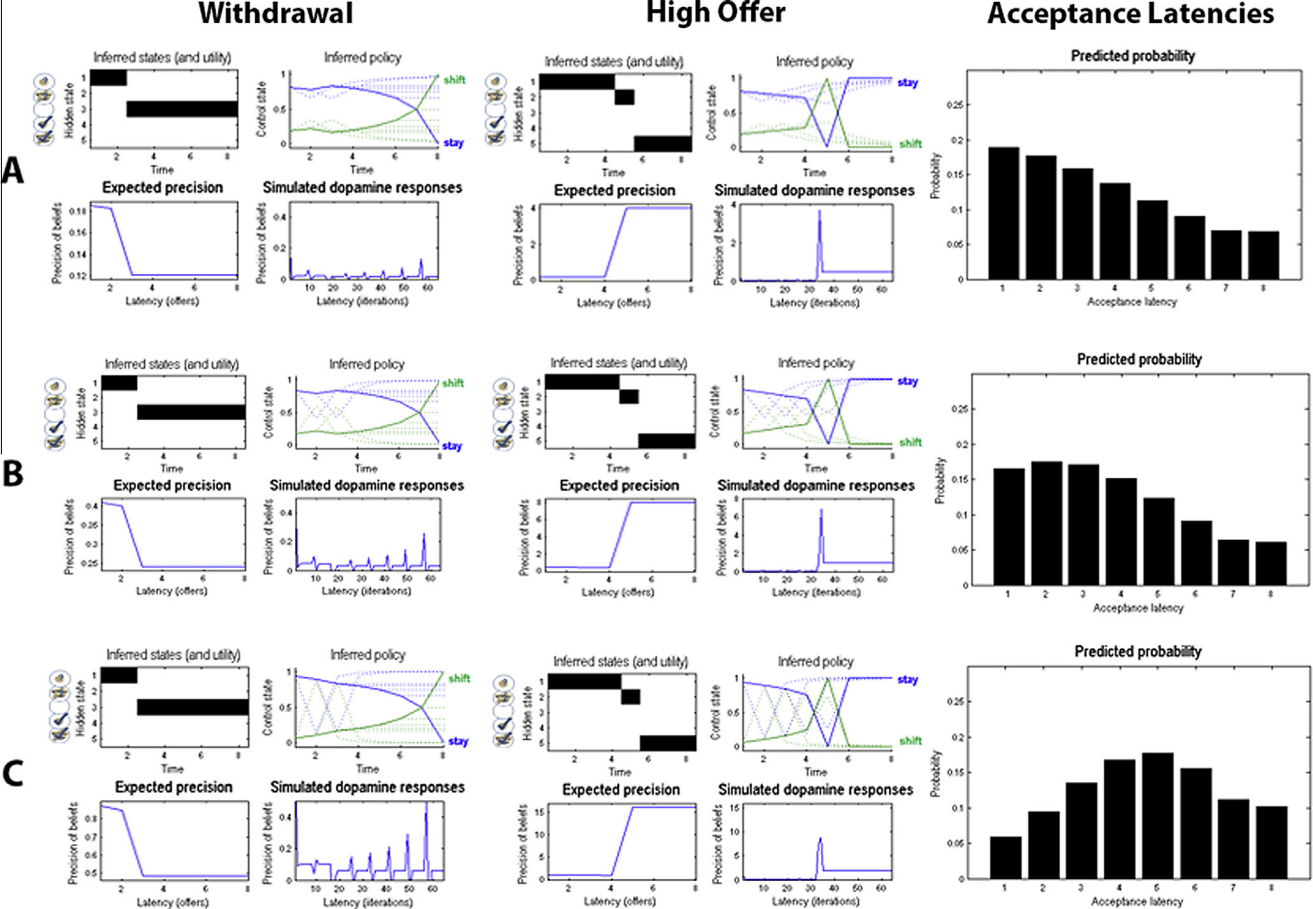
Effects of precision on behaviour. (A–C) reflect low (*α* = 4), medium (*α* = 8) and high (*α* = 16) prior precision, respectively. The left column shows expectations about latent states and precision in an example game with initial offer withdrawal at trial three. In each panel, the top row shows the inferred hidden state the agent believes this is in (upper left) and the expected probability of accepting or declining as a function of trials (upper right panel: dotted lines for earlier trials and the solid lines for the last trial). These expectations are updated using expected precision that is shown as a function of trial in the lower row – in terms of the precision at the end of each trial (lower left) and simulated dopamine responses reflecting the changes in precision (lower right). See [69] for further details about these (variational) updates. Here, the initial propensity to wait (stay) depends on prior precision and increases for higher values of precision (upper right panel). Intuitively, the drop of expected precision when the initial offer is withdrawn is larger with higher prior precision. The middle column shows the equivalent updates when a high offer is presented in the fifth trial. Expected precision displays a sharp increase when the high offer is received, where the size of the increase again depends on individual prior precision. The right column shows the observed acceptance probabilities at each time step, simulated in 256 games. It becomes obvious that the acceptance latencies are shifted towards later trials with increasing precision (confidence that the high offer will be received).

Precision reflects the confidence about reaching a desired goal; i.e., the successful implementation of a policy. In active inference, this means that precision reflects the degree of stochasticity or goal-directedness of behaviour. In the context of the limited offer task, precision reflects confidence about receiving the high offer. Thus, subjects with a higher prior precision tend to wait longer for the high offer and accept the initial offer less often – which can be understood as being more confident that the high offer will eventually transpire. Conversely, subjects with a low precision will display more variability in their behaviour and are less confident about waiting for the high offer. Consequently, they will be more impulsive in accepting the initial offer. It is important to appreciate that precision can account for this type of ‘reflection impulsivity’ (i.e., a tendency to respond spontaneously with little deliberation) but not for higher risk-taking: if precision increases, subjects will be very sensitive to differences in the values of policies (e.g., waiting until a later trial before accepting the initial offer) and act accordingly. In other words, they will not deviate much from their favoured policy over successive games. This means that increasing precision will not compel subjects to wait too long and become too risk-seeking, it will just make them more sensitive to the advantages of planned and goal-directed behaviour. Conversely, decreasing precision will make subjects less confident in receiving the high offer and thus less goal-directed. This represents a state of poor self-control, causing the subject to display habitual behaviour. Subjects, therefore, will accept the initial offer too early because they cannot postpone their response – and therefore show a lack of inhibitory control (impulsive behaviour). Fig. 4 shows the effects of prior precision on behaviour when the offer is withdrawn or accepted as well as the propensity to accept the initial offer (safe option) simulated in 256 games for different prior expectations about precision.

A possible mechanism underlying impulsivity – and a lack of inhibitory control – could therefore be lower prior precision, reflecting more stochastic (inconsistent) behaviour and less confidence in the successful implementation of long-term policies (and therefore less goal-directedness). This provides an explanation for why some subjects accept earlier than would be optimal for gain maximisation in this task [69]. Note that Schwartenbeck et al. [69] were able to show that precision, as defined here, is encoded in the dopaminergic midbrain, which fits comfortably with the association between impulsive behaviour and dysfunction of the dopaminergic system in addiction.

**Fig. 5:**
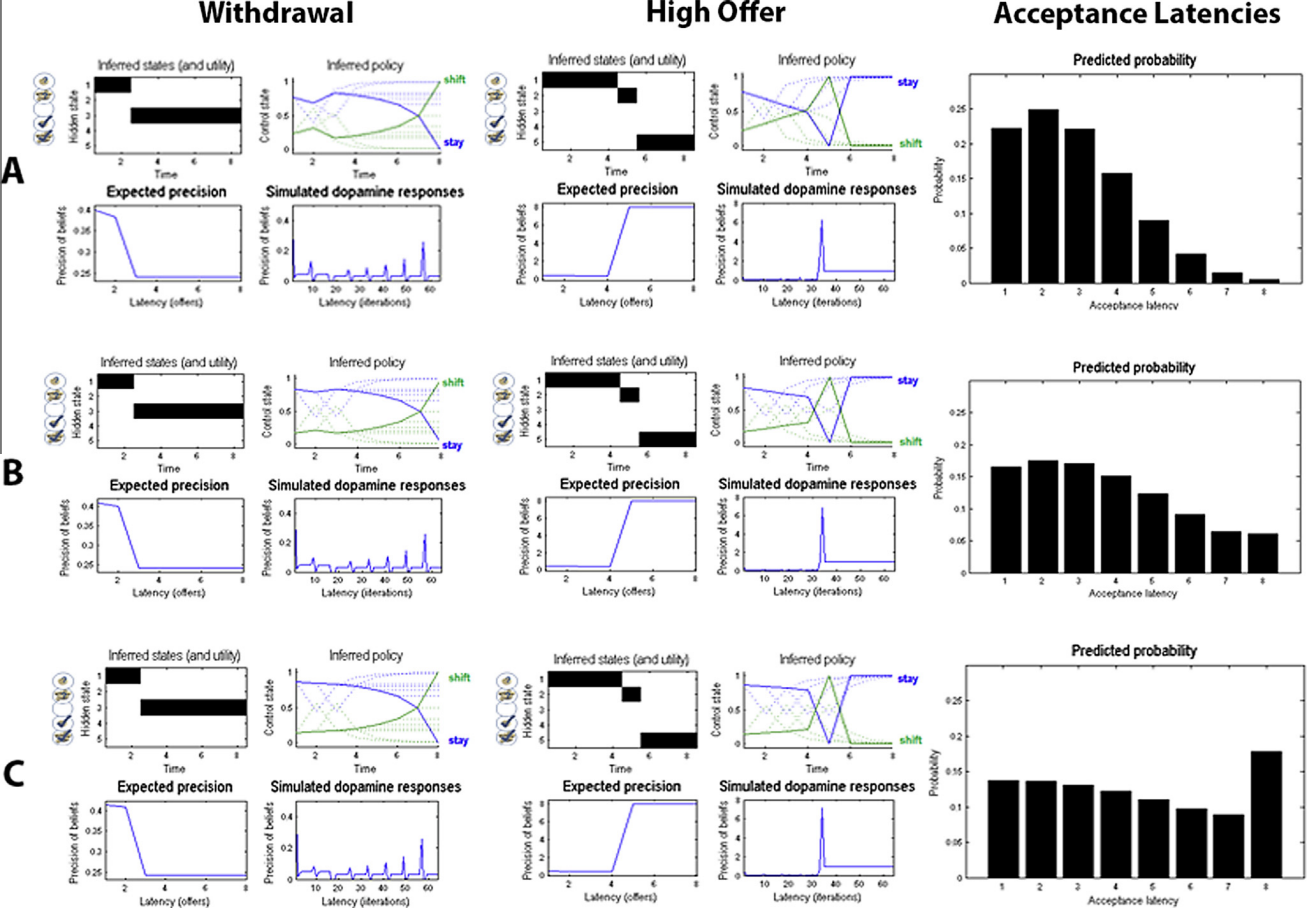
Effects of estimates of the hazard rate on behaviour. (A–C) reflect high (losing the initial offer is likely), medium and low (losing the initial offer is unlikely) estimates of the hazard rate, respectively. This figure uses the same format as Fig. 4: the left and middle column show expectations about hidden states, behaviour and expected precision for a withdrawal of the initial offer (at the third trial) and the receipt of a high offer at the fifth trial, respectively. This time, the behaviour and initial level of precision is identical, but the expected behaviour reflects the differences in hazard rates – with the probability of waiting being smaller if losing the initial offer is likely. The right column predicts that subjects will accept the initial offer early if a loss is thought to be likely.

However, we also know that higher risk-taking – often associated with steeper discounting of future rewards or events – is a key characteristic of addictive decision-making. In the context of our limited offer task, being too risk-seeking would imply that subjects wait too long for the high offer – and thus risk losing the initial offer. How could this be represented in a subject’s generative model? As discussed earlier, subjects also have to represent the hazard rate of a withdrawal and the receipt of a high offer. Being too optimistic about winning the high offer – without considering the danger of losing the initial offer – could be explained by a poor expectation of the hazard rate, such that one assumes that receiving the high offer is very likely whereas losing the initial offer is unlikely. In contrast to precision, the individual representation of the hazard rate can account for higher risk-taking or (equivalently) the overconfidence in winning regardless of the risks, a common phenomenon in pathological gambling [21,20]. Fig. 5 illustrates the effects of adopting different hazard rates.

**Fig. 6:**
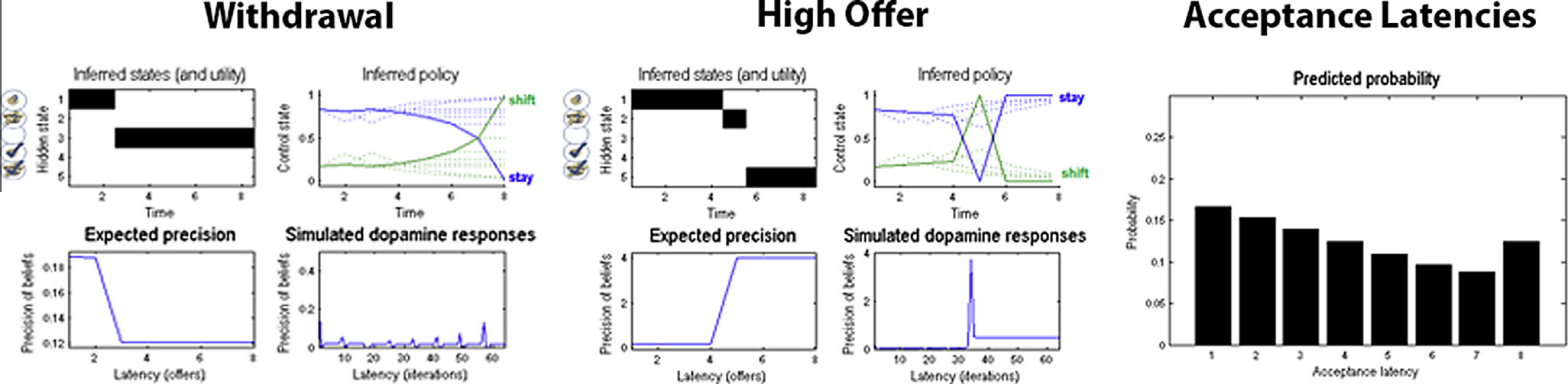
Effects of a low prior precision and a low estimate of the hazard rate on behaviour. Low precision causes subjects to be more stochastic and less patient in their behaviour, whereas a low estimate of the hazard rate causes subjects to become more risk-seeking in waiting for the high offer. See legends for Figs. 4 and 5 for details.

Note that a suboptimal representation of the hazard rate also explain the converse; namely, accepting prematurely because one estimates that a withdrawal is very likely whereas receiving the high offer is not likely at all. However, this form of being overly cautious is not consistent with previous findings of choice behaviour in addiction, which is marked by higher impulsivity, less goal-directed behaviour and higher risk-taking. One might therefore hypothesise that people suffering from addiction will exhibit lower prior precision, causing behaviour to be more impulsive and stochastic, combined with a lower prior expectation about the hazard rate, inducing higher risk-taking and lower risk-sensitivity, as illustrated in Fig. 6. By investigating the prior expectations in a group of substance-based addiction, behavioural addiction and a control group one should be able to characterise general differences and commonalities on an individual and group level – and associate these differences in particular parameters with differences in observed behaviour and neuronal activation.

## Discussion

We have argued that in order to understand inter-individual variability – and pathological behaviour – it is necessary to move from a descriptive or normative account of optimality to a definition of optimal inference, based on an individual’s generative model of the world. People are now starting to appreciate that casting atypical behaviour (e.g., planning) as inference does not necessarily imply apparent flaws in an inference process, but calls for a deeper understanding of biologically plausible approximations or bounds on probabilistic inference [2,26,65,72]. To illustrate this point we have used a generic model of choice behaviour, which provides a formal characterisation of decision processes in terms of prior expectations causing observed behaviour. We have tried to substantiate this approach by using it to simulate a potential behavioural phenotype of addiction in a ‘Limited offer task’.

These simulations also provide a nice illustration of how a computational account of pathologic behaviour can provide the basis for understanding similar behaviour that is caused by different mechanisms. As discussed earlier, obsessive compulsive disorders (OCD) have been associated with habitual and impoverished goal directed behaviour, but may be caused by high anxiety rather than high reflection impulsivity (i.e., a lack of confidence or precision) [33,34,44]. Both Figs. 4A and 5A show behaviour that is less goal-directed – and marked by a propensity to accept an initial offer in early trials – as one would expect to see in addiction and patients with OCD. In the former case, however, overly habitual behaviour is caused by reduced confidence or high impulsivity; i.e., the inability to decline a current offer (also marked by higher dispersion over acceptance latencies). In the latter simulation, the short acceptance latency is caused by pessimistic prior expectations about the hazard rate; in other words, assuming that a withdrawal is very likely. This could be induced by overly cautious or anxious (pessimistic) expectations, and thus might explain the predominance of habitual behaviour in OCD. In short, characterising the generative model underlying suboptimal behaviour provides a principled approach to understanding the origins of maladaptive behaviour as well as the diverse computational phenotypes that present similar ‘symptoms’. This further illustrates the diagnostic value of a computational approach to psychiatry. Casting choice behaviour as (active) Bayesian inference may provide a useful tool to do so – and thus help improve our diagnostic assessment and evaluation of therapeutic success.

Note that our scheme allows us to identify likely causes that underlie pathologic behaviour – such as prior expectations that causes behavioural and neuronal responses in addiction. It neither makes strong predictions about the development of an addiction nor was it designed to be (*a priori*) sensitive to addictive choice behaviour. Rather, the framework provides a generic account of decision-making that allows one to assess the individual (generative) model that underlies (Bayes optimal) choice, and thus the individual characteristics that underlie addictive choice. Crucially, this approach posits specific mechanisms that may impair decision-making in addiction. This may be relevant for differentiating addiction from other conditions and may also – more practically – prove useful for assessing the current status of an addict (and their therapeutic progress) or for predicting relapse.

In conclusion, we believe that an active (Bayesian) inference framework may be useful for quantifying maladaptive choice behaviour in addiction, such as being too impulsive and risk seeking or showing a predominance of habitual behaviour when making choices. Having specified our hypotheses and theoretical considerations, we look forward to presenting empirical tests of these ideas in the near future.

## Conflict of interest

The authors declare no conflict of interest.
