# Corrigendum to ‘Optimal inference with suboptimal models: Addiction and active Bayesian inference’ [Med. Hypotheses 84 (2015) 109–117]

**DOI:** 10.1016/j.mehy.2016.02.021

**Published:** 2016-06

**Authors:** Philipp Schwartenbeck, Thomas H.B. FitzGerald, Christoph Mathys, Ray Dolan, Friedrich Wurst, Martin Kronbichler, Karl Friston

**Affiliations:** a The Wellcome Trust Centre for Neuroimaging, UCL, 12 Queen Square, London WC1N 3BG, UK; b Centre for Cognitive Neuroscience, University of Salzburg, Salzburg, Austria; c Neuroscience Institute, Christian-Doppler-Klinik, Paracelsus Medical University Salzburg, Salzburg, Austria; d Department of Psychiatry and Psychotherapy II, Christian-Doppler Hospital, Paracelsus Medical University, Salzburg, Austria; e Max Planck UCL Centre for Computational Psychiatry and Ageing Research, London WC1B 5EH, UK

The authors regret to have omitted the affiliation of the Max Planck UCL Centre for Computational Psychiatry and Ageing Research.

Furthermore, the authors regret to have erroneously specified the second affiliation as ‘Institute for Cognitive Neuroscience’, which should be the ‘Centre for Cognitive Neuroscience’.

The authors would like to apologise for any inconvenience caused.

